# Sodium Thiosulfate in Acute Myocardial Infarction: A Randomized Clinical Trial

**DOI:** 10.1016/j.jacbts.2023.06.001

**Published:** 2023-08-23

**Authors:** Marie-Sophie L.Y. de Koning, Paulien van Dorp, Solmaz Assa, Gabija Pundziute-Do Prado, Michiel Voskuil, Rutger L. Anthonio, Duco Veen, Tim Leiner, Anita J. Sibeijn-Kuiper, Harry van Goor, Dirk J. van Veldhuisen, Peter van der Meer, Robin Nijveldt, Erik Lipšic, Pim van der Harst

**Affiliations:** a Department of Cardiology, University of Groningen, University Medical Centre Groningen, Groningen, the Netherlands; b Department of Cardiology, Division of Heart and Lungs, University Medical Centre Utrecht, Utrecht, the Netherlands; c Department of Cardiology, Treant Hospital, location Scheper, Emmen, the Netherlands; d Department of Methodology and Statistics, Utrecht University, Utrecht, the Netherlands; e Optentia Research Programme, North-West University, Vanderbijlpark Campus, Vanderbijlpark, South Africa; f Department of Radiology, University Medical Centre Utrecht, Utrecht, the Netherlands; g Department of Radiology, Mayo Clinic, Rochester, Minnesota, USA; h University of Groningen, University Medical Centre Groningen, Cognitive Neuroscience Centre, Groningen, the Netherlands; i Department of Pathology and Medical Biology, University of Groningen, University Medical Centre Groningen, Groningen, the Netherlands; j Department of Cardiology, Radboud University Medical Centre, Nijmegen, the Netherlands

**Keywords:** clinical trial, hydrogen sulfide, ischemia-reperfusion injury, myocardial infarction, randomized controlled trial, thiosulfates

## Abstract

In this proof-of-principle trial, the hypothesis was investigated that sodium thiosulfate (STS), a potent antioxidant and hydrogen sulfide donor, reduces reperfusion injury. A total of 373 patients presenting with a first ST-segment elevation myocardial infarction received either 12.5 g STS intravenously or matching placebo at arrival at the hospital and 6 hours later. The primary outcome, infarct size, measured by cardiac magnetic resonance at 4 months after randomization, did not differ between the treatment arms. Secondary outcomes were comparable as well, suggesting no clinical benefit of STS in this population at relatively low risk for large infarction.

## Highlights

• This proof-of-concept trial aimed to investigate the potential protective effect of STS, an antioxidant and H_2_S donor, against ischemia-reperfusion injury in patients presenting with STEMI.
• A total of 373 patients with a first STEMI were randomly assigned to receive either STS or matching placebo at presentation and 6 hours thereafter.
• Overall myocardial infarct size determined by cardiac magnetic resonance after 4 months was 8.4%. Treatment with STS was well tolerated, but did not result in a reduction in infarct size.

## Visual Abstract

**Figure:**
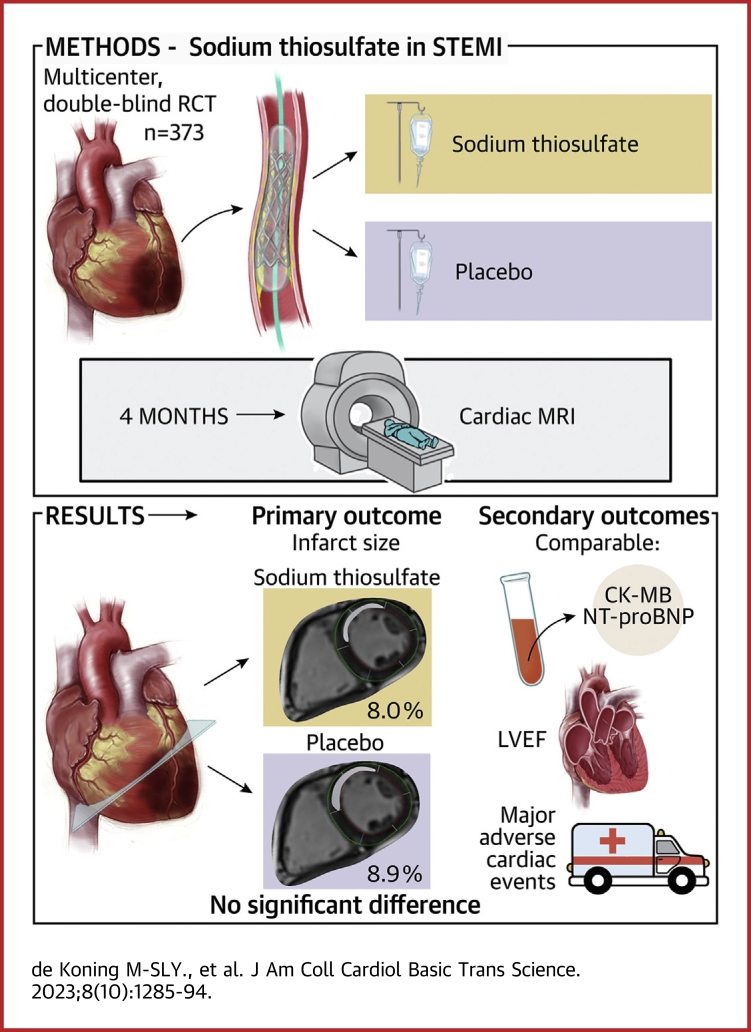

Myocardial infarction is a common cause of disability and death worldwide.^1^ Infarct size is the major determinant for the future development of heart failure and reduced life expectancy.^2^ Major progress has been made to limit infarct size, mainly by thrombolysis and primary percutaneous coronary intervention (PCI) in combination with antiplatelet therapy. Despite this progress, death rate, heart failure, and recurrent cardiac events continue to remain substantial in patients presenting with acute myocardial infarction.^3^^,^^4^

Reperfusion of ischemic myocardium can paradoxically induce myocardial injury, and experimental data suggest that this injury can contribute up to 50% of the final myocardial infarct size.^5^, ^6^, ^7^, ^8^, ^9^ Myocardial reperfusion injury is a therapeutic target for which currently no effective treatment exists, and the search for an effective therapy is ongoing.^8^^,^^10^, ^11^, ^12^

Sodium thiosulfate (STS) is a strong antioxidant and anti-inflammatory compound with vasoactive properties.^13^ Moreover, as a donor of the physiological gaseous signaling molecule hydrogen sulfide (H_2_S), it might also exert additional H_2_S-related beneficial effects, including the maintenance of mitochondrial integrity, vasodilatation, activation of antiapoptotic and antioxidant pathways, and anti-inflammatory effects.^14^, ^15^, ^16^, ^17^ STS is clinically used for the treatment of acute cyanide poisoning, calciphylaxis, carbon monoxide toxicity, and cisplatin toxicities in cancer therapy.^18^, ^19^, ^20^, ^21^ H_2_S donating compounds, including STS, have also been successful in reducing reperfusion injury in a wide variety of preclinical models.^13^^,^^22^, ^23^, ^24^, ^25^, ^26^ In an earlier dose-escalation pilot study, we demonstrated that STS was well tolerated in patients presenting with an acute coronary syndrome undergoing PCI.^27^

In this proof-of-principle trial, we tested the hypothesis that STS treatment reduces infarct size in patients presenting with ST-segment elevation myocardial infarction (STEMI).

## Methods

### Trial design and oversight

GIPS-IV (Groningen Intervention Study for the Preservation of Cardiac Function with STS after STEMI) is an investigator-driven, randomized, controlled, double blind trial conducted in 3 high-volume PCI centers in the Netherlands: University Medical Centre Groningen, Groningen, the Netherlands; University Medical Centre Utrecht, Utrecht, the Netherlands; and Treant Hospital, Emmen, the Netherlands. Details of the trial design have been previously published.^28^ All study procedures were in accordance with the Declaration of Helsinki and Good Clinical Practice guidelines. The conduct of the trial was supervised by the trial steering committee. An independent data and safety monitoring board (DSMB) oversaw the safety of the trial. The authors designed and coordinated the trial, oversaw the study conduct and reporting, managed the database, and wrote all drafts of the paper. All of the authors vouch for the accuracy and completeness of the reported data and analyses. The contents of this paper are consistent with the research protocol, which was approved by the ethics committee (Ref. 2016.381; Groningen, the Netherlands) and national authority. Before commencing enrollment, the trial was registered in a clinical trial registry under number (NCT02899364). Detailed information about the trial organization is available in Supplemental Appendix.

### Study population

Adults who presented with a first STEMI were eligible if their symptoms started within 12 hours before presentation and their symptoms were ongoing and/or ST-segment elevation was persistent upon arrival at the cardiac catheterization laboratory. Patients with a history of prior myocardial infarction, coronary artery bypass grafting or cardiomyopathy, a malignancy treated with chemotherapy and/or radiotherapy (chest region), or any condition that did not allow the patient to successfully undergo cardiac magnetic resonance (CMR) or participate in the study were excluded. Details of the inclusion and exclusion criteria are provided in the trial protocol.

### Trial procedures

The study procedures were designed not to delay primary PCI. Immediately after arrival at the cardiac catheterization laboratory, witnessed oral consent was obtained by the interventional cardiologist, and patients directly received the next-in-sequence randomized kit of either STS 12.5 g or matching placebo. Study medication was dissolved in 250 mL of normal saline and administered intravenously in 20 to 30 minutes before and during PCI. Six hours after the first dose, a second dose of study medication was administered. The rationale behind the timing and dosage of study medication has been previously published.^28^ Known side effects of STS include nausea, vomiting, and hypotension,^20^ which were specifically monitored before and after each dose of study medication. Due to high incidences of nausea and vomiting observed during the execution of the trial, the DSMB recommended to preventively administer antiemetics (metoclopramide 10 mg intravenously) before each dose of study medication. The trial protocol was amended accordingly. During hospitalization, written informed consent was obtained, and creatine kinase MB (CK-MB) was measured to determine enzymatic infarct size. Four months after randomization, participants were scheduled for a hospital visit to obtain CMR and to assess adverse events and N-terminal pro–B-type natriuretic peptide (NT-proBNP). In case a participant declined CMR, adverse events were assessed by telephone. Study medication was produced, randomized, and labelled according to Good Manufacturing Practice by A15 Pharmacy. Randomization was performed in a 1:1 ratio in permuted blocks of 4, with stratification by recruiting site and for anterior vs nonanterior myocardial infarction. The patient, interventional cardiologist, all caregivers, data collectors, and the CMR core laboratory were blinded to treatment allocation.

### Trial outcomes

CMR is the preferred method for the identification of potential benefits associated with new cardioprotective strategies.^29^ The primary outcome of this trial was infarct size expressed as percentage of LV mass. Infarct size was measured 4 months after randomization, a time in which the infarct healing is expected to be completed,^30^ and infarct size no longer changes substantially.^31^, ^32^, ^33^ Secondary outcome parameters included the effect of STS on peak CK-MB during index hospitalization, left ventricular ejection fraction (LVEF) on CMR at 4 months, and NT-proBNP concentration at 4 months follow-up. Clinical events were also assessed up to 4 months after randomization and included all-cause mortality, the combined incidence of major adverse cardiovascular events (cardiovascular death, reinfarction, unscheduled reintervention), stent thrombosis, stroke, implantable cardioverter-defibrillator implantation, and hospitalization for chest pain or heart failure. All potential clinical endpoints were adjudicated by an independent endpoint adjudication committee blinded to treatment allocation. The endpoint definitions are available in Supplemental Methods II.

### CMR protocol

All CMR studies were performed on a 3-T clinical MR scanner (multivendor Siemens, Philips), using a phased array cardiac receiver coil. Electrocardiogram-gated balanced steady-state free precession cine images were acquired during repeated breath holds in the standard long-axis views (4-, 3-, and 2-chamber views) and contiguous short-axis slices covering the entire left ventricle. Using identical slice locations, late gadolinium enhanced images were acquired at least 10 minutes after intravenous administration of a gadolinium-based contrast agent (0.2 mmol/kg) with a single shot inversion recovery gradient-echo pulse sequence. The epicardial and endocardial borders were outlined in end-systolic and -diastolic images to measure left ventricular volumes and calculate LVEF. Infarct size was quantified using an automated method (full width at half maximum) with manual correction.^34^^,^^35^ All CMR scans were evaluated by an independent core laboratory (Radboud UMC) using dedicated software (QMass, Medis Suite 3.2.28.0). The core laboratory was blinded to treatment allocation and clinical patient data. All CMR measurements and calculations were performed, and data was locked before unblinding.

### Statistical analysis

This trial was designed as a proof-of-concept study. We considered a relative reduction of 33% in infarct size relevant.^33^ In the previous GIPS-III trial, the mean infarct size was 9.0% ± 7.9%.^36^ To have 85% power to detect a 3% absolute difference in change in infarct size between the STS and placebo group, we calculated that 125 patients would need to be enrolled in each group, assuming a 2-sided α of 0.05. Based on experience from local and previous studies, we allowed for up to a 33% dropout for the primary endpoint caused by contraindications for CMR—eg, implantable cardioverter-defibrillator implantation, claustrophobia, or unable to obtain sufficient image quality for infarct size detection.^36^, ^37^, ^38^, ^39^ Therefore, we expected to need to include 380 patients to obtain a reliable primary outcome measure in 250 patients. Due to the COVID-19 pandemic, the actual drop-out for the CMR visit after 4 months was higher than anticipated, but this did not result in the recommendation of the DSMB or ethical committee to extend enrollment.

Baseline characteristics are summarized as mean ± SD or median with 25th and 75th percentiles (Q1-Q3) depending on data distribution. Categorical variables are displayed as count and percentages. All analyses were performed according to a prespecified analysis plan that was finalized before database lock and unblinding. No formal interim analysis took place. Missing data were not imputed.

The primary outcome, infarct size, was analyzed with Beta regression on an intention-to-treat basis. Treatment allocation, recruiting site, and anterior myocardial infarction were added to the model as fixed variables. The regression coefficient for treatment allocation is the primary outcome and is reported as the (marginal average) difference in infarct size between the STS and placebo group, together with a *P* value and 95% confidence interval (CI). As described in the design paper,^28^ we used Beta regression for the primary analysis because it is the preferred method of analysis for proportional data with a non-normal distribution. Likewise, a Beta regression within the per-protocol population was performed, including all patients who received complete treatment with study medication, without major protocol deviations. Irrespective of the primary outcome reaching statistical significance, prespecified subgroup analyses were performed using regression analyses with a test for interaction for age (below vs above the median), gender, TIMI (Thrombolysis In Myocardial Infarction) flow pre-PCI (≤1 vs >1), infarct location (anterior vs nonanterior myocardial infarction), ischemic time (below vs above the median), single vs multivessel disease, and the time from start of study medication to first coronary intervention (below vs above the median).

For analyses of secondary outcomes, when binary, treatment comparisons were performed using the Fisher exact or chi-square test. For continuous outcomes, independent samples Student’s *t*-tests or Mann-Whitney *U* tests were used, as appropriate.

A 2-sided α of 0.05 was considered statistically significant. Analyses were performed with STATA version 14.0 (StataCorp).

## Results

From July 16, 2018, through March 2, 2021, a total of 1,650 patients presenting with suspected STEMI in 1 of the 3 recruiting centers were screened for eligibility (Supplemental Figure 1). A total of 380 patients underwent randomization and received a first dose of study medication. Seven patients withdrew informed consent resulting in a study population of 373 participants: 186 participants assigned to the STS group and 187 to the placebo group. The characteristics of the patients were well balanced in the 2 treatment groups at baseline (Tables 1 and 2) and at discharge (Supplemental Table 1). The mean age in the overall population was 62 ± 12 years, and 23.1% of the patients were women. The median time from onset of complaints to wire passage was 141 minutes (Q1, Q3: 102, 177 minutes). Before primary PCI, 371 (99.5%) patients received aspirin, and all patients received a loading dose of a P2Y_12_ inhibitors and heparin. TIMI 0 or 1 flow before PCI was observed in 245 (65.7%) patients, and the left anterior descending artery was identified as the culprit lesion in 152 (40.8%). After PCI, a TIMI flow grade 0 or 1 was observed in 14 (3.8%) patients.

**Table 1:** Characteristics of the Patients at Baseline

	STS (n = 186)	Placebo (n = 187)
Demography		
Age at randomization, y	62.3 ± 11.5	61.8 ± 12.0
Male	140 (75.3)	147 (78.6)
Body mass index, kg/m^2^	27.3 ± 4.0	27.1 ± 4.6
Caucasian ethnicity	181 (97.3)	182 (97.3)
Prior conditions		
Hypertension	86 (46.2)	82 (43.9)
Dyslipidemia	66 (35.5)	67 (35.8)
Current smokers	73 (39.2)	71 (38.0)
Positive family history	79 (42.7)	80 (42.8)
Diabetes mellitus	23 (12.4)	28 (15.0)
Previous MI	5 (2.7)	1 (0.5)
Previous PCI	4 (2.2)	1 (0.5)
Clinical characteristics		
Systolic blood pressure, mm Hg	138 ± 25	143 ± 26
Diastolic blood pressure, mm Hg	84 ± 17	86 ± 16
Heart rate, beats/min	73 ± 16	75 ± 17
Killip class I	171 (96.6)	180 (96.8)
Laboratory parameters		
Hemoglobin, mmol/L	8.6 (8.1, 9.2)	8.7 (8.0, 9.3)
Creatinine, μmol/L	75 (65, 86)	75 (64, 86)
CK, U/L	127 (82, 211)	134 (90, 232)
CK-MB activity, U/L	15 (12, 20)	16 (13, 23)
NT-proBNP, ng/L	106 (40, 221)	87 (43, 216)
Glucose, mmol/L	5.7 (5.5, 6.1)	5.6 (5.5, 6.1)

Values are mean ± SD, n (%), or median (Q1, Q3). Baseline characteristics stratified by treatment allocation.

CK = creatine kinase; CK-MB = creatine kinase-myocardial band; MI = myocardial infarction; NT-proBNP = N-terminal pro–B-type natriuretic peptide; PCI = percutaneous coronary intervention; Q1, Q3 = 25th, 75th percentile.

**Table 2:** Procedural Characteristics

	STS (n = 186)	Placebo (n = 187)
Time from symptom onset to start study medication, min	119 (80, 193)	131 (90, 215)
Time from symptom onset to wire passage, min	133 (97, 203)	147 (104, 233)
Single-vessel disease	103 (55.4)	91 (48.7)
Culprit territory		
Left anterior descending	76 (40.9)	76 (40.6)
Circumflex or marginal	29 (15.6)	27 (14.4)
Right coronary artery	77 (41.4)	77 (41.2)
Left main	0 (0.0)	1 (0.5)
No clear culprit	4 (2.2)	6 (3.2)
Medication from first medical care to PCI		
Aspirin	186 (100)	185 (98.9)
Loading dose of P2Y_12_	186 (100)	187 (100)
Heparin	186 (100)	187 (100)
Glycoprotein IIb/IIIa inhibitor	29 (15.6)	33 (17.6)
TIMI flow grade pre-PCI		
0	114 (61.3)	111 (59.4)
1	10 (5.4)	10 (5.3)
2	29 (15.6)	22 (11.8)
3	32 (17.2)	43 (23.0)
Cannot be defined	1 (0.5)	1 (0.5)
Proximal lesion	77 (41.4)	77 (41.2)
Initial intervention of culprit lesion		
PCI	181 (97.3)	176 (94.1)
CABG	1 (0.5)	3 (1.6)
Conservative	4 (2.2)	8 (4.3)
No reflow observed on angiography	5 (2.8)	7 (4.0)
Distal embolization after PCI	16 (8.8)	10 (5.7)
TIMI flow grade post-PCI		
0	5 (2.8)	4 (2.3)
1	4 (2.2)	1 (0.6)
2	3 (1.7)	10 (5.7)
3	169 (93.4)	161 (91.5)

Values are median (Q1, Q3) or n (%).

CABG = coronary artery bypass graft; TIMI = Thrombolysis In Myocardial Infarction; other abbreviations as in Table 1.

### Primary outcome

CMR was completed in 238 patients 4 months after randomization (median 4.0 months [Q1, Q3: 3.8, 4.5 months], full range 3.4 to 8.2 months). The primary outcome parameter, infarct size on CMR, could be determined in 116 patients in the STS group and 110 in the placebo group (Supplemental Figure 1). The baseline, procedural, and discharge characteristics were also well balanced between the STS and placebo-treated patients of the CMR population (Supplemental Tables 2 to 4).

**Figure 1:**
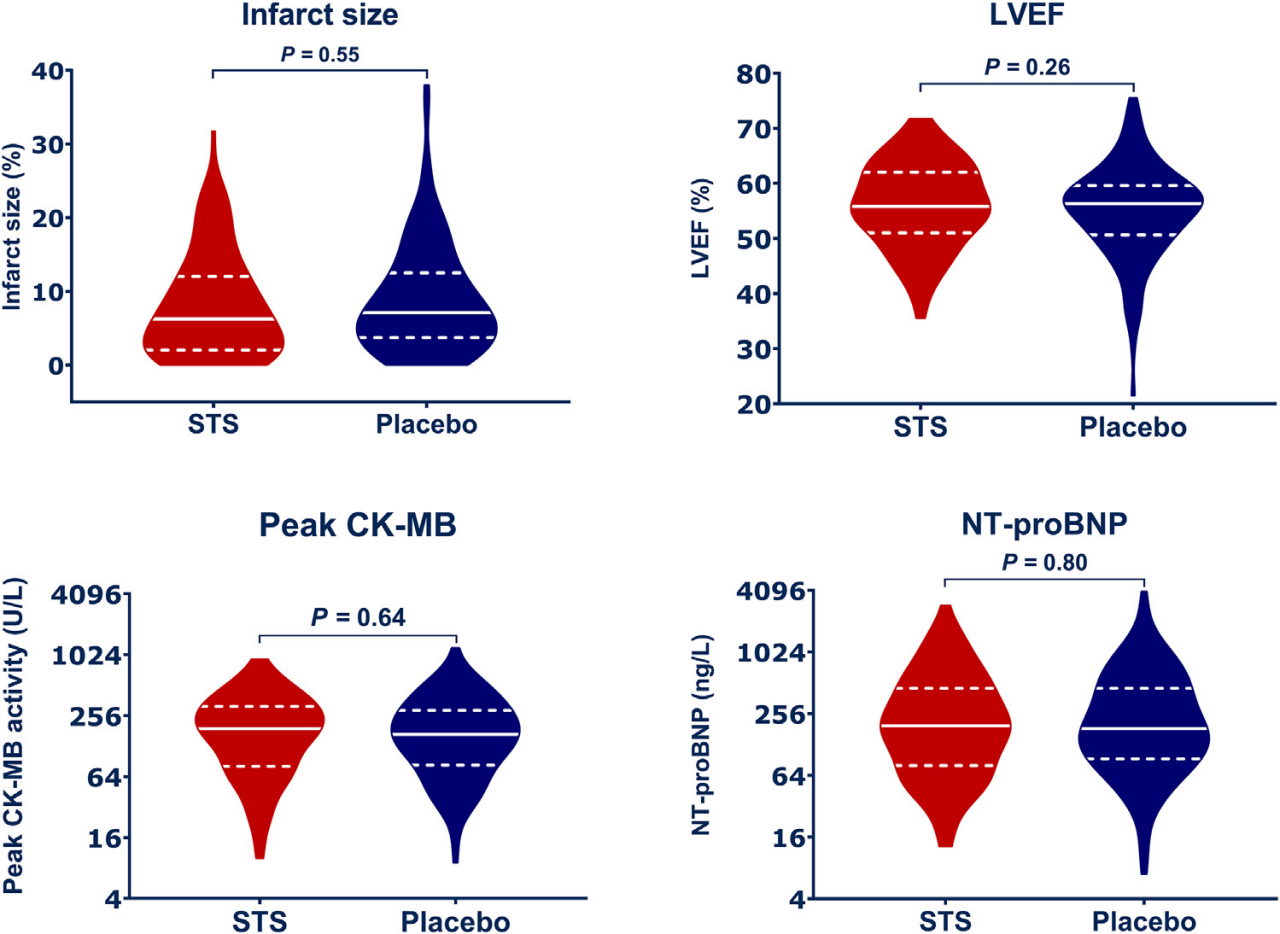
Primary and Secondary Outcomes by Allocated Treatment Violin plots showing medians (solid line) and 25th and 75th percentiles (dashed line) for the primary outcome, infarct size at 4 months follow-up, and secondary outcomes in patients treated with sodium thiosulfate (STS) (red) and patients treated with placebo (navy). No significant differences were observed between treatment arms, suggesting no clinical benefit of STS in this relatively low-risk study population. CK-MB = creatine kinase myocardial band; LVEF = left ventricular ejection fraction; NT-proBNP = N-terminal pro–B-type natriuretic peptide.

Infarct size at 4 months after randomization did not differ between the STS and placebo-treated patients. Mean infarct size in the STS group was 8.0% ± 7.0% and was 8.9% ± 7.4% in the placebo group. The marginal average change in infarct size in the STS group was −0.6%; 95% CI: −2.4% to 1.2%; *P =* 0.55, compared with participants treated with placebo (Figure 1, Table 3).

**Table 3:** Outcomes and Clinical Events at 4 Months

	STS	Placebo	*P* Value
Primary outcome			
Infarct size (% of LV mass)	8.0 ± 7.0	8.9 ± 7.4	0.55^a^
Secondary outcomes			
Peak CK-MB, U/L^b^	191 (81, 315)	168 (84, 289)	0.64
Left ventricular ejection fraction, %	56.1 ± 7.6	54.9 ± 8.7	0.26
NT-proBNP, ng/L	195 (80, 452)	183 (97, 445)	0.80
Clinical endpoints^c^			
Major adverse cardiovascular events^d^	6 (3.2)	11 (5.9)	0.22
Cardiovascular mortality	1 (0.5)	2 (1.1)	0.57
Noncardiovascular mortality	1 (0.5)	0 (0.0)	0.32
Recurrent myocardial infarction	3 (1.6)	9 (4.8)	0.08
STEMI	2 (1.1)	6 (3.2)	0.16
NSTEMI	1 (0.5)	3 (1.6)	0.32
Recurrent revascularization	4 (2.2)	5 (2.7)	0.74
Target-lesion revascularization	3 (1.6)	3 (1.6)	0.99
Target-vessel revascularization	0	0	
Non–target-vessel revascularization	1 (0.5)	2 (1.1)	0.57
CABG	0	0	
Stent thrombosis	2 (1.1)	3 (1.6)	0.66
Stroke	1 (0.5)	0 (0.0)	0.32
Hospitalization for heart failure	0 (0.0)	1 (0.5)	0.32
Hospitalization for chest pain	6 (3.2)	3 (1.6)	0.31
ICD implantation	0	0	

Values are mean ± SD, median (Q1, Q3), or n (%).

CABG = coronary artery bypass graft surgery; ICD = Implantable cardioverter-defibrillator; NSTEMI = non–ST-segment elevation myocardial infarction; STEMI = ST-segment elevation myocardial infarction; STS = sodium thiosulfate; other abbreviations as in Table 1.

a Analyzed with Beta Regression adjusted for site, treatment, and anterior MI location.

b Results only shown for CK-MB activity (University Medical Centre Groningen), other sites measured CK-MB mass (n = 43); these results were consistent with results for CK-MB activity (data not shown).

c Definitions are available in the Supplemental Appendix.

d Cardiovascular death, reinfarction, reintervention.

### Secondary outcomes

Enzymatic infarct size was available for 248 patients: 124 in the STS and 124 in the placebo group. Missing values were caused by early transfer to referring hospital or hemolytic sample. The median peak creatine kinase MB value was 191 U/L (Q1, Q3: 81, 315 U/L) in the STS group compared with 168 U/L (Q1, Q3: 84, 289 U/L) in the placebo group *(P =* 0.64) (Figure 1, Table 3). LVEF at 4-month follow-up was determined by CMR in 230 patients. The average LVEF was 56.1% ± 7.6% in the STS group compared with 54.9% ± 8.7% in the placebo group *(P =* 0.26). NT-proBNP at 4 months was also comparable for both treatment strata (Figure 1, Table 3).

### Clinical endpoints

At 4 months after randomization, no patients were lost to follow-up. Four patients died: 2 in the STS group and 2 in the placebo group. The combined incidence of cardiovascular death, reinfarction, and unplanned revascularization at 4 months was 3.2% (n = 6) in the STS group and 5.9% (n = 11) in the placebo group *(P =* 0.22). The rates of all other clinical endpoints, including stroke, cerebrovascular accident, and hospitalization for heart failure or chest pain, are presented in Table 3.

### Treatment-related adverse effects

Patients in the STS group were more likely to experience nausea and vomiting than those in the placebo group (Table 4). Nausea and vomiting continued to occur more frequently in the STS group after the standard use of antiemetics before administration of study medication. A 23 ± 23 mm Hg decline in systolic blood pressure was observed after administration of the first dose of study medication in both treatment arms *(P =* 0.55). Blood pressure remained constant after the second dose of study medication in both groups. Other adverse effects were mild and transient. No severe adverse events were observed that were considered to be related to STS treatment.

**Table 4:** Adverse Effects

	STS	Placebo	*P* Value
Serious adverse events, total number	18 (9.7)	18 (9.6)	0.99
Patients with an adverse event	122 (65.6)	132 (70.6)	0.30
Adverse events of special interest			
First dose			
New-onset nausea	40 (21.7)	11 (5.9)	<0.001
New-onset vomiting	25 (13.7)	4 (2.2)	<0.001
Without preventive antiemetics			
New-onset nausea	25 (33)	8 (12)	0.002
New-onset vomiting	13 (17)	2 (3)	0.005
With preventive antiemetics			
New-onset nausea	15 (13.9)	3 (2.6)	0.002
New-onset vomiting	12 (11.2)	2 (1.7)	0.004
Second dose			
New-onset nausea	31 (18.8)	6 (3.6)	<0.001
New-onset vomiting	18 (10.9)	2 (1.2)	<0.001
Without preventive antiemetics			
New-onset nausea	14 (19)	2 (3)	0.001
New-onset vomiting	7 (9)	1 (1)	0.024
With preventive use antiemetics			
New-onset nausea	17 (19)	4 (4)	0.002
New-onset vomiting	11 (12)	1 (1)	0.003

Values are n (%).

STS = sodium thiosulfate.

### Per-protocol analysis and subgroup analyses

**Figure 2:**
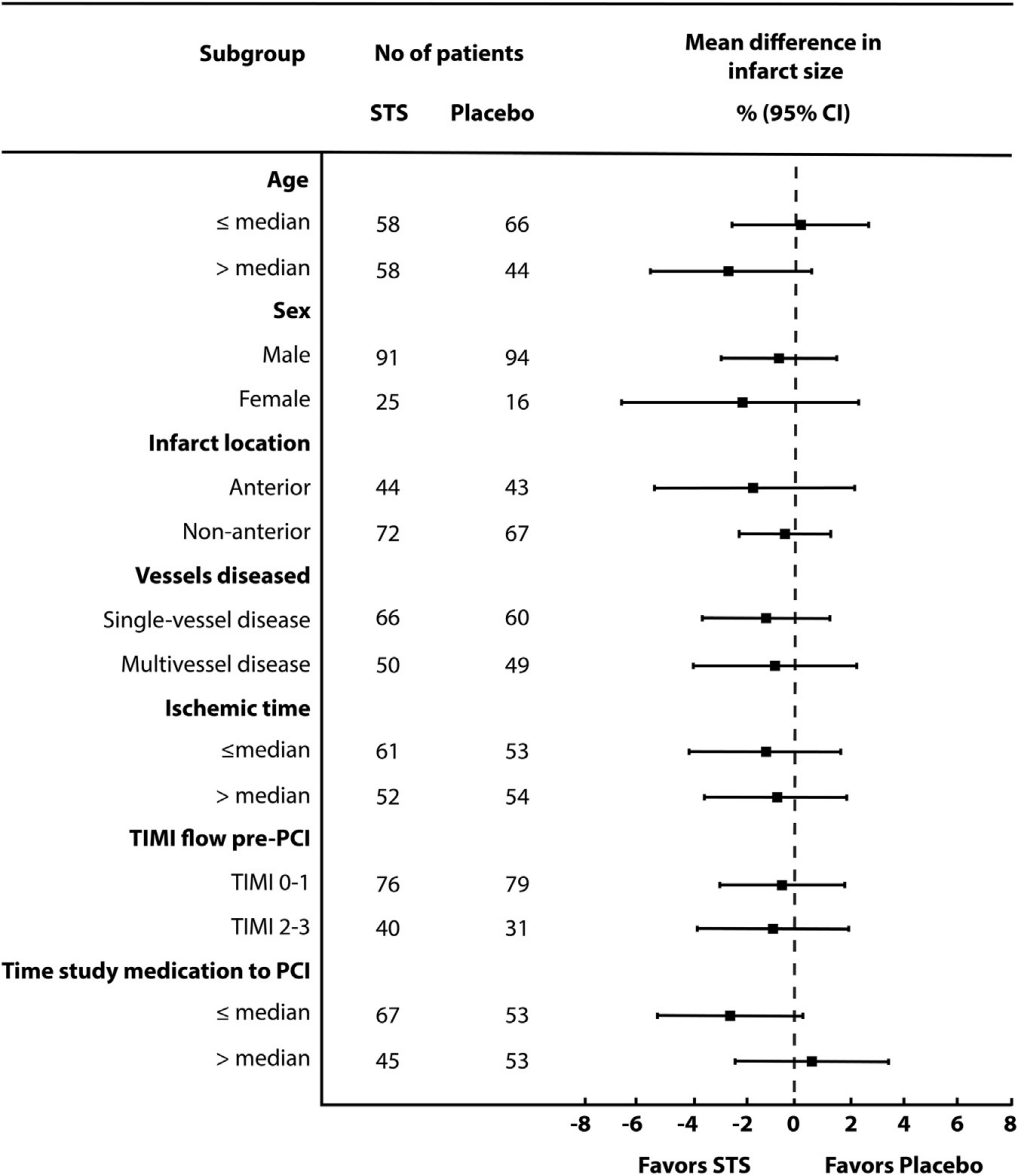
Effect of Sodium Thiosulfate on Infarct Size Across Prespecified Subgroups Forest plot depicting the estimated treatment effect of sodium thiosulfate on the primary outcome infarct size at 4-month follow-up across prespecified subgroups (age ≤ median [61 years] vs age > median; male vs female; anterior myocardial infarction vs nonanterior myocardial infarction; singe-vessel disease vs multivessel disease; ischemic time ≤ median [141 minutes] vs > the median; TIMI flow pre-PCI 0-1 vs. 2-3; and time from start of study medication to PCI ≤ median [16 minutes] vs > the median). Treatment effects of STS were consistent across all subgroups (*P* for interaction all >0.05). PCI = percutaneous coronary intervention; STS = sodium thiosulfate: TIMI = Thrombolysis In Myocardial Infarction.

The results of the per-protocol analysis were consistent with the intention to-treat analysis (Supplemental Table 5). The results of the primary endpoint were also consistent across prespecified subgroups (Figure 2).

## Discussion

Among patients presenting with STEMI, intravenous STS treatment initiated before primary PCI did not reduce myocardial infarct size compared with placebo. There was also no effect on LVEF. Patients in the STS group were more likely to experience nausea and vomiting than those in the placebo group.

Patients presenting with STEMI are routinely treated with primary PCI to treat myocardial ischemia with reperfusion. Reperfusion to an ischemic area has been associated with cellular injury, which may substantially contribute to the final infarct size.^8^^,^^10^ The current proof-of-concept study was developed to clinically translate the plethora of preclinical studies providing strong mechanistic and functional evidence that STS and H_2_S can substantially reduce reperfusion injury.^14^^,^^40^ For example, substantial infarct sparing effects were observed in both small and large animal models using different H_2_S sources.^15^^,^^24^^,^^40^, ^41^, ^42^, ^43^, ^44^

STS has also been shown to be beneficial in several other clinical settings associated with cellular toxicity (eg, cyanide intoxication, calciphylaxis, reduction of cisplatin-related toxicity). In our study, STS induced the known side-effects such as nausea and vomiting, but did not reduce myocardial infarct size.

Several findings may explain the lack of benefit of STS in our patient cohort. First, the final infarct size of our patients was relatively small. This is a consequence of the well-organized STEMI network in the Netherlands with short ischemic time (2-2.5 hours vs 3 hours in other recent studies)^45^, ^46^, ^47^ and pretreatment by the ambulance service with a loading dose of a P2Y_12_ inhibitor, aspirin, and intravenous heparin. Moreover, we also included patients with TIMI 2 to 3 flow pre-PCI, because the study treatment was initiated before performing the coronary angiogram to reach therapeutic levels before reperfusion, bearing in mind that early administration of study medication might increase the likelihood of cardioprotection,^48^ and that the majority of detrimental effects of ischemia-reperfusion injury already occur during the first moments after reperfusion.^8^ Potential benefit of STS cannot be excluded in the absence of pretreatment with antiplatelets or in a certain subgroup of patients—eg, those presenting with completely closed arteries for extended time, combined with high Killip class, and large area at risk, who thus are patients with higher probability of possible additional myocardial salvage.^49^ Also, in setting of low availability or unavailability of primary PCI, resulting in delayed reperfusion, STS might reduce myocardial injury. Second, the required cardioprotective concentrations of STS might be higher than could be achieved in this trial. However, the dosage was based on prior efficacy data in humans and was limited by the known side effects.^20^^,^^50^^,^^51^ Furthermore, the amount of STS and H_2_S released in the heart during reperfusion remains unknown. Future substudies in stored blood samples might provide insight in concentrations and effects on oxidative stress and inflammation. Finally, the duration of treatment was limited to the first hours after reperfusion (T_1/2_ ≈ 3 hours), whereas reperfusion injury lasts longer.^21^^,^^52^ Development of oral preparations might enable continued treatment for an extensive period of time, potentially allowing the reported anti-inflammatory, antioxidant, and proangiogenic properties of H_2_S/STS, to modify outcomes.^25^^,^^53^ Finally, failure to translate preclinical studies into clinical benefit might originate from the absence of comorbidities and comedications in animal models.^49^

The incidence of adverse side effects, mainly nausea and vomiting, was comparable to STS use in other conditions.^19^^,^^20^ The emetogenic effect of STS did not result in discontinuation of study medication, and the additional use of prophylactic antiemetic agents, as was recommended by the DSMB, appeared to reduce the incidence of nausea and vomiting. Potential effects of antiemetics on cardioprotection could not be ruled out.^54^ However, the preemptive administration in both treatment groups minimized this potential bias. The change in blood pressure that we observed in both groups after the first dose of study medication was likely caused by administration of vasodilators, required for the radial PCI procedure, because between-group differences were not observed and no change in blood pressure occurred after the second dose of study medication.

### Study limitations

First, partly because of the national COVID-19 pandemic restrictions to visit the hospital for non-essential care and fear of patients to acquire a COVID-19 infection, the actual percentage of patients who underwent randomization who were available for the primary outcome measure was 59% instead of the anticipated 66%. This led to a reduction of statistical power from the desired 85% to the actual 80%. Post-hoc, it seems unlikely that adding ∼10 more participants to each arm would have substantially modified our findings. Also, the studied number of patients with CMR remains in line with recommendations for the evaluation of cardioprotective strategies.^29^ Second, our study was also not powered to detect clinical outcomes such as all-cause mortality or hospitalization for heart failure. However, CMR-determined infarct size has been the recommended primary outcome for early assessment of potential cardioprotective therapies.^29^ The relevance of our primary outcome is also supported by the reported strong graded response with subsequent mortality and hospitalization for heart failure.^2^ We did not take into account area at risk when determining infarct sizes. However, the comparable percentages of proximal culprit lesions (also within each culprit vessel) in both treatment arms (41%) suggest balanced areas at risk. Finally, women were under-represented, and very few patients were non-Caucasian.

## Conclusions

We assessed the effect of STS in a proof-of-concept study of patients with STEMI undergoing primary PCI. The administration of STS at time of reperfusion did not lead to a reduction in infarct size. The studied STS dose was not associated with significant adverse events.

Perspectives **COMPETENCY IN MEDICAL KNOWLEDGE:** In this proof-of-concept study, administration of the hydrogen sulfide donor STS did not reduce infarct size, as measured by cardiac magnetic resonance, in patients presenting with acute STEMI. STS was also not associated with important harmful effects.

**TRANSLATIONAL OUTLOOK:** Future clinical trials in populations at higher risk for large myocardial infarction may potentially uncover clinical benefit from STS or other hydrogen sulfide-donating compounds.

## Funding Support and Author Disclosures

This study is supported by a grant of the Netherlands Organization for Health Research and Development and the Dutch Heart Foundation (ZonMW; project No: 95105012), Siemens health care GmbH (Push project IPA No.10), and the University Medical Centre Groningen. The subsidizers had no role in the design and conduct of the study, study analyses, drafting or editing of the manuscript, and its final contents. Dr Anthonio has received a Biotronic Teaching Grant, a Sanofi CTCue license for 1 year, and payment for Abiomed Impella Webcast; and Amgen paid for attendance at the New York Cardiovascular Symposium 2019. Dr van der Meer has received consultancy fees and/or grants from Novartis, Novo Nordisk, Vifor Pharma, AstraZeneca, Pfizer, Pharmacosmos, Pharma Nord and Ionis. Dr Nijveldt has received unrestricted research grants from Biotronik and Philips; and has received speaker fees from Sanofi Genzyme and Bayer. Dr Lipsic has received an educational grant from Abbott Medical. All other authors have reported that they have no relationships relevant to the contents of this paper to disclose.
