# Supplementary material for: Sodium Thiosulfate in Acute Myocardial Infarction: A Randomized Clinical Trial
Source: JACC Basic Transl Sci. 2023 Aug 23;8(10):1285–94. doi: 10.1016/j.jacbts.2023.06.001 (PMC10714171; doi:10.1016/j.jacbts.2023.06.001)
Supplement: Supplemental Material [file mmc1.pdf]

**Supplemental Material**

*Sodium Thiosulfate in Acute Myocardial Infarction: a Randomized Clinical Trial*


| | **Page** |
|---|---|
| Supplemental Methods I - Trial organization | 2 |
| Supplemental Methods II - Definitions clinical endpoints | 5 |
| Supplemental Figure 1 Trial flowchart | 12 |
| Supplemental Table 1 Medication at discharge | 13 |
| Supplemental Table 2 Baseline characteristics of the CMR population | 14 |
| Supplemental Table 3 Procedural characteristics of the CMR population | 15 |
| Supplemental Table 4 Medication at discharge in the CMR population | 16 |
| Supplemental Table 5 Per-protocol analysis | 17 |

**Supplemental Methods I – Trial organization**

**GIPS-IV investigators**

University Medical Center Groningen, Groningen, the Netherlands.

M.L.Y. de Koning MD PhD; P. van Dorp MD; H.W. van der Werf MD; M.G. Dickinson MD PhD; G. Pundziute-Do Prado MD PhD; A.F.M. van den Heuvel MD PhD; E.S. Tan MD PhD; J.J. Wykrzykowska MD PhD; P.J.J. Vlaar MD PhD; R.A.J. Schurer MD; G.E.H. Leenders MD PhD; W.T. Ruifrok MD PhD; L.A. Teeuwen MD; H.E. Groot MD PhD; D. Ties MD PhD; R. van Dijk MD PhD; M.H.T. Hartman MD PhD; T. Hendriks MD PhD; C.G. Maagdenberg MD; J.M. ter Maaten MD PhD; S. Assa MD PhD; M.H. Wiertsema MD; Y.T. Klip MD PhD; P. van der Meer MD PhD; Dirk J van Veldhuisen MD PhD; N.W. Smit PhD; B. Dorhout[†] PhD; E. Lipsic MD PhD; P. van der Harst MD PhD from the department of Cardiology. H. van Goor PhD from the Department of Pathology and Medical Biology. HH Boersma PharmD PhD; TYJ Appeldoorn PharmD; E.L. Bergsma PharmD from the department of Pharmacy. J. Jongsma from the echo core lab; Y.M. Hummel PhD from the echo core lab and Us2.ai.

Treant Hospital, location Scheper, Emmen, the Netherlands.

D. Ketelaar; L. Schaafsma; S.E. van der Kooi; H. Giezen; W.T. Ruifrok MD PhD; P. van der Vleuten MD PhD; G.A. Jessurun[†] MD PhD; A. Schramm MD PhD; S.A. Klein MD PhD; S.H.K. The MD PhD; R.L. Anthonio, MD PhD from the department of Cardiology. J. Engelhart PharmD from the department of Pharmacy.

University Medical Center Utrecht, Utrecht, the Netherlands

L.M. Frerichs-van Doleweerd; K. Vlaardingerbroek; M.J. Kuikhoven; A.O. Kraaijeveld MD PhD; Z.H. Rittersma MD PhD; P.R. Stella MD PhD; G.J. Vlachojannis MD PhD; P. van der Harst MD PhD; M. Voskuil MD PhD from the department of Cardiology. C. de Goede from the department of Pharmacy.

**CMR imaging centers**

University Medical Center Groningen, Groningen, the Netherlands.

A.J. Sibeijn-Kuiper; R.J. Renken PhD from the Cognitive Neuroscience Center; G Pundziute-Do Prado MD PhD from the department of Cardiology; R. Vliegenthart MD PhD from the department of Radiology.

University Medical Center Utrecht, Utrecht, the Netherlands

T. Leiner MD PhD, Department of Cardiology & Mayo Clinic.

**CMR core laboratory**

Radboud UMC, Nijmegen, the Netherlands

R. Nijveldt MD PhD from the Department of Cardiology.

**Steering committee**

P. van der Harst MD PhD (chair), Department of Cardiology, University Medical Center Groningen, Groningen, the Netherlands & Department of Cardiology, University Medical Center Utrecht

E. Lipsic MD PhD, Department of Cardiology, University Medical Center Groningen, Groningen, the Netherlands

M.L.Y. de Koning MD PhD, Department of Cardiology, University Medical Center Groningen, Groningen, the Netherlands

S. Assa MD PhD, Department of Cardiology, University Medical Center Groningen, Groningen, the Netherlands & Department of Cardiology, Maastricht University Medical Center, Maastricht, The Netherlands

R.J. Renken PhD, University of Groningen, University Medical Center Groningen, Cognitive Neuroscience Center, Groningen, The Netherlands

D. Veen PhD, Department of Methodology and Statistics, Utrecht University, Utrecht, the Netherlands & Optentia Research Programme, North-West University, Vanderbijlpark Campus, Vanderbijlpark, South Africa.

**Data and Safety Monitoring Board**

K.C.B. Roes PhD, Department of Biostatistics, Radboud UMC, Nijmegen, the Netherlands & chair methodology of the Dutch Medicines Evaluation Board.

H. Boersma Ir PhD, Department of Cardiology Erasmus Medical Center Rotterdam, the Netherlands & Member of the Dutch Medicines Evaluation Board;

E. Kedhi MD PhD, Department of Cardiology, St Jan hospital, Bruges, Belgium;

J.M. ten Berg MD PhD, Department of Cardiology, Antonius hospital, Nieuwegein, the Netherlands & professor in Interventional Cardiology at University of Maastricht, Maastricht, the Netherlands.

**Endpoint Adjudication Committee**

T.N.E. Vossenberg MD, Department of Cardiology, Medical Center Leeuwarden, Leeuwarden, the Netherlands;

V.E. Hagens MD PhD, Department of Cardiology, Ommelander hospital Groningen, Scheemda, the Netherlands;

M.A.H. van Leeuwen MD PhD, Department of Cardiology, Isala Clinics, Zwolle, the Netherlands.

**Users' committee**

R. de Jong, G-cure BV, Groningen, the Netherlands;

D.C. Sagel, University Medical Center Groningen Ambulance care, Groningen, the Netherlands;

H.H. Boersma PharmD PhD, Department of Pharmacy, University Medical Center Groningen, Groningen, the Netherlands;

H.J. Hektor PhD, Technology Transfer desk, University Medical Center Groningen, Groningen, the Netherlands;

J. Leenders, Dutch Heart foundation, The Hague, the Netherlands;

H. Doornkamp, Haren GN, the Netherlands;

P. van der Harst MD PhD, University Medical Center Groningen, Groningen, the Netherlands & University Medical Center Utrecht, Utrecht, the Netherlands.

**Writing committee**

University Medical Center Groningen, Groningen, the Netherlands

P. van der Harst MD PhD, University Medical Center Groningen, Groningen, the Netherlands & University Medical Center Utrecht, Utrecht, the Netherlands.

E. Lipsic MD PhD, University Medical Center Groningen, Groningen, the Netherlands.

M.L.Y. de Koning MD PhD University Medical Center Groningen, Groningen, the Netherlands.

**Trial statistics**

University Medical Center Utrecht, Utrecht, the Netherlands

D.E. Grobbee MD PhD, Julius Centre for Health Sciences and Primary Care, University Medical Center Utrecht, Utrecht, the Netherlands.

D. Veen PhD, Department of Methodology and Statistics, Utrecht University, Utrecht, the Netherlands & Optentia Research Programme, North-West University, Vanderbijlpark Campus, Vanderbijlpark, South Africa.

D. Cianci PhD, Julius Centre for Health Sciences and Primary Care, University Medical Center Utrecht, Utrecht, the Netherlands & methodology assessor for the Dutch Medicines Evaluation Board.

**Data monitoring and data management**

Schutjens Clinical Research Consultancy, Zwinderen, the Netherlands

G.A.M. Schutjens

**Supplemental Methods II – Definitions clinical endpoints**

## 1. Mortality

The EAC will adjudicate all subject deaths. Death will be adjudicated for cardiac and all cause death. All deaths are considered cardiac unless an unequivocal non-cardiac cause can be established. Specifically, any unexpected death even in patients with coexisting potentially fatal non-cardiac disease (e.g. cancer, infection) will be classified as cardiac death.

*Cardiac death*

Cardiac death will be adjudicated for any death due to proximate cardiac cause (eg, MI, low-output failure, fatal arrhythmia), unwitnessed death and death of unknown cause, and all procedure related deaths, including those related to concomitant treatment. A subdivision in cardiac death is made, namely heart failure, sudden cardiac death and other.

Sudden cardiac death is either defined as witnessed, un-witnessed, cardiac arrest without evidence of circulatory collapse, such as hypotension, exacerbation of congestive heart failure, or altered mental status, before the disappearance of the pulse or abrupt collapse occurring within one hour of the onset of the symptoms that resulted in death.

Death due to heart failure will be defined as death due to clinically end-stage heart failure during hospital admission or by exacerbation of congestive heart failure reported by an attending general practitioner.

For all these deaths, no probable non-cardiac cause should be suggested by the history or autopsy;

*Non cardiac death*

Any death not covered by the above definitions, such as death caused by infection, malignancy, sepsis, pulmonary causes, accident, suicide, or trauma.

## 2. Myocardial infarction

The EAC will adjudicate all cases of MI, after the initial index (STEMI) event defined by the documented fall of cardiac markers, and the relationship of the event to the target/culprit vessel and the presence of stent-thrombosis. All infarcts that cannot be clearly attributed to a vessel other than target/culprit vessel will be considered related to the target vessel.

Criteria for Acute Myocardial Infarction (based on the Fourth Universal Definition of Myocardial Infarction Guidelines, ESC 2018):

The term acute myocardial infarction should be used when there is acute myocardial injury with clinical evidence of acute myocardial ischemia with detection of rise and/or fall of cardiac troponin (cTn) values with at least one value above the 99th percentile of the upper reference limit (URL) , together with at least one of the following:

• Symptoms of myocardial ischemia;

• ECG changes indicative of new ischemia (new ST-T changes or new left bundle branch block [LBBB]);

• Development of pathological Q waves in the ECG;

• Imaging evidence of new loss of viable myocardium or new regional wall motion abnormalities in a pattern consistent with an ischemic etiology;

• Identification of an intracoronary thrombus by angiography or autopsy (not for types 2 or 3 MIs).

Post-mortem demonstration of acute athero-thrombosis in the artery supplying the infarcted myocardium meets criteria for *type 1 MI*.

Evidence of an imbalance between myocardial oxygen supply and demand unrelated to acute athero-thrombosis meets criteria for *type 2 MI*.

• Cardiac death in patients with symptoms suggestive of myocardial ischemia and presumed new ischemic ECG changes, but death occurred before cTn were obtained, or before cTn values would be increased meets criteria for (type 3) MI.

Coronary procedure related MI (type 4a and 5)

• Percutaneous coronary interventions (PCI) related MI (≤48 hours after the procedure) is arbitrarily defined by elevation of cTn value > 5 X 99th percentile URL in patients with normal baseline values.

Patients with elevated pre-procedural cTn values, in whom the pre-procedural cTn levels are stable (≤20% variation) or falling, must meet a >5 fold increase and manifest a change from the baseline value of >20%. In addition, either (1) new ischemic ECG changes or (2) development of new pathological Q waves or (3) angiographic findings consistent with a procedural flow-limiting complication or (4) imaging evidence of loss of viable myocardium that is presumed to be new and in a pattern consistent with an ischemic etiology.

• Coronary artery bypass grafting (CABG) related MI is arbitrarily defined by elevation of cTn value (> 10 X 99th percentile URL) in patients with normal baseline values Patients with elevated pre/procedural cTn values, in whom the pre-procedural cTn levels are stable (≤20% variation) or falling, must meet a >10 fold increase and manifest a change from the baseline value of >20%. In addition, either (1) development of new pathological Q waves or (2) angiographic findings consistent with a procedural flow-limiting complication such as occlusion of a major epicardial graft or (3) imaging evidence of loss of viable myocardium that is presumed to be new and in a pattern consistent with an ischemic etiology.

Isolated development of new pathological Q waves meets the *type 4a MI* or *type 5 MI* criteria with either revascularization procedure if cTn values are elevated and rising but less than the pre-specified thresholds for PCI and CABG.

Other types of 4 MI include *type 4b MI* stent thrombosis and *type 4c MI* restenosis that both meet *type 1 MI* criteria.

Post-mortem demonstration of a procedure-related thrombus meets the *type 4a MI* criteria or *type 4b MI* criteria if associated with a stent.

*STEMI/NSTEMI*

Myocardial infarction will be subdivided into ST-elevated myocardial infarction and non ST-elevated myocardial infarction.

*STEMI*

To meet criteria for STEMI, acute chest pain and persistent (>20 min) ST-segment elevation (measured at the J-point) on the electrocardiogram should be present in at least two contiguous leads with ST-segment elevation ≥ 2.5 mm in men < 40 years, ≥2 mm in men ≥ 40 years, or ≥ 1.5 mm in women in leads $V_2$–$V_3$ and/or ≥ 1 mm in the other leads [in the absence of left ventricular (LV) hypertrophy or left bundle branch block (LBBB)].

*NSTEMI*

Patients with NSTEMI should present with acute chest pain but no persistent ST-segment elevation. ECG changes may include transient ST-segment elevation, persistent or transient ST-segment depression, T-wave inversion, flat T waves or pseudo-normalization of T waves or the ECG may be normal.

**3. Stent thrombosis**

The EAC will adjudicate all cases of stent thromboses for confirmation.

*Definite stent thrombosis*

Angiographic confirmation of stent thrombosis

The presence of a thrombus that originates in the stent or in the segment 5 mm proximal or distal to the stent and presence of at least 1 of the following criteria within a 48-hour time window:

Acute onset of ischemic symptoms at rest

New ischemic ECG changes that suggest acute ischemia

Typical rise and fall in cardiac biomarkers (refer to definition of spontaneous MI)

Non-occlusive thrombus: Intracoronary thrombus is defined as a (spheric, ovoid, or irregular) non calcified filling defect or lucency surrounded by contrast material (on 3 sides or within a coronary stenosis) seen in multiple projections, or persistence of contrast material within the lumen, or a visible embolization of intraluminal material downstream.

Occlusive thrombus: TIMI 0 or TIMI 1 intra-stent or proximal to a stent up to the most adjacent proximal side branch or main branch (if originates from the side branch).

Pathological confirmation of stent thrombosis

Evidence of recent thrombus within the stent determined at autopsy or via examination of tissue retrieved following thrombectomy.

*Probable stent thrombosis*

Clinical definition of probable stent thrombosis is considered to have occurred after intracoronary stenting in the following cases:

Any unexplained death within the first 30 days, irrespective of the time after the index procedure,

Any MI that is:

related to documented acute ischemia in the territory of the implanted stent without angiographic confirmation of stent thrombosis, and in the absence of any other obvious cause

*Possible stent thrombosis*

Clinical definition of possible stent thrombosis is considered to have occurred with any unexplained death from 30 days after intracoronary stenting until end of trial follow-up. We follow the ARC definitions. Stent Thrombosis should be reported as a cumulative value at the different time points and with the different separate timepoints. Time 0 is defined as the time point after the guiding catheter has been removed and the patient left the Cath lab.

*Timing*

Acute stent thrombosis: 0 to 24 hours after stent implantation

Subacute stent thrombosis: 24 hours to 30 days after stent implantation

Late stent thrombosis: 30 days to 1 year after stent implantation

Very late stent thrombosis: 1 year after stent implantation

**4. Revascularization**

Any repeat revascularization procedure (PCI or CABG) will be considered as an event, except for scheduled revascularization based on the index CAG to diagnose and treat coronary artery lesions

identified during the index procedure and heart team discussion. In case a scheduled revascularization based on the index CAG to treat coronary artery lesions identified during the index procedure will be performed earlier than originally scheduled due to an urgent medical reason (for example ongoing chest pain) it will be adjudicated as recurrent revascularization. In case the scheduled revascularization is performed earlier without urgent medical reason, it will not be adjudicated as a recurrent revascularization.

*Target Lesion Revascularization (TLR)*

TLR is defined as a repeat percutaneous intervention of the target/culprit lesion of the index CAG. The target lesion is defined as the treated segment from 5 mm proximal to the stent and to 5 mm distal to the stent.

*Target Vessel Revascularization (TVR)*

TVR is defined as a repeat percutaneous intervention in the same coronary artery as the index procedure (which includes upstream and downstream branches), but not in the same coronary segment (defined as the treated segment from 5mm proximal to the stent and 5 mm distal to the stent).

*Non Target Vessel Revascularization (NTVR)*

Any repeat percutaneous intervention not covered by the above definitions for target lesion and target vessel revascularization.

*Coronary artery bypass grafting*

New therapeutic coronary bypass grafting after the index procedure.

**5. Cerebrovascular accident**

Acute neurological event of at least 24 hours of duration, with focal signs and symptoms and without evidence supporting any alternative explanation. Diagnosis of stroke requires confirmation by CT or MRI or pathological confirmation. Stroke is further classified as ischemic, hemorrhagic or type

uncertain. Ischemic stroke occurs as a result of an obstruction within a blood vessel supplying blood to the brain. Hemorrhagic stroke includes intraparenchymal, subarachnoid hemorrhage and subdural hematomas.

**6. Hospitalization**

Hospitalization is defined as an non-elective admission to the hospital after discharge from the index event with overnight stay (different dates for admission and discharge).

*Hospitalization for heart failure*

Signs and symptoms consistent with heart failure, confirmed by clinical findings and laboratory parameters, and;

No alternative clinical explanation for the signs and symptoms.

*Hospitalization for chest pain*

Recurrent signs and symptoms suggestive of ischemia, and;

Absence of new or recurrent ST-segment elevations or depression suggestive of MI, and;

No rise of biochemical markers of myocardial necrosis (including troponin, CK-Mb, CK) to above the upper limit of normal (or if markers already elevated, greater than 50% of the lowest recovery enzyme level from the index infarction), and;

No alternative clinical explanation for the signs and symptoms.

**7. ICD-implantation**

Implantation of an Internal Cardiac Defibrillation after the index event.

**Supplemental Figure 1 - Trial flowchart**

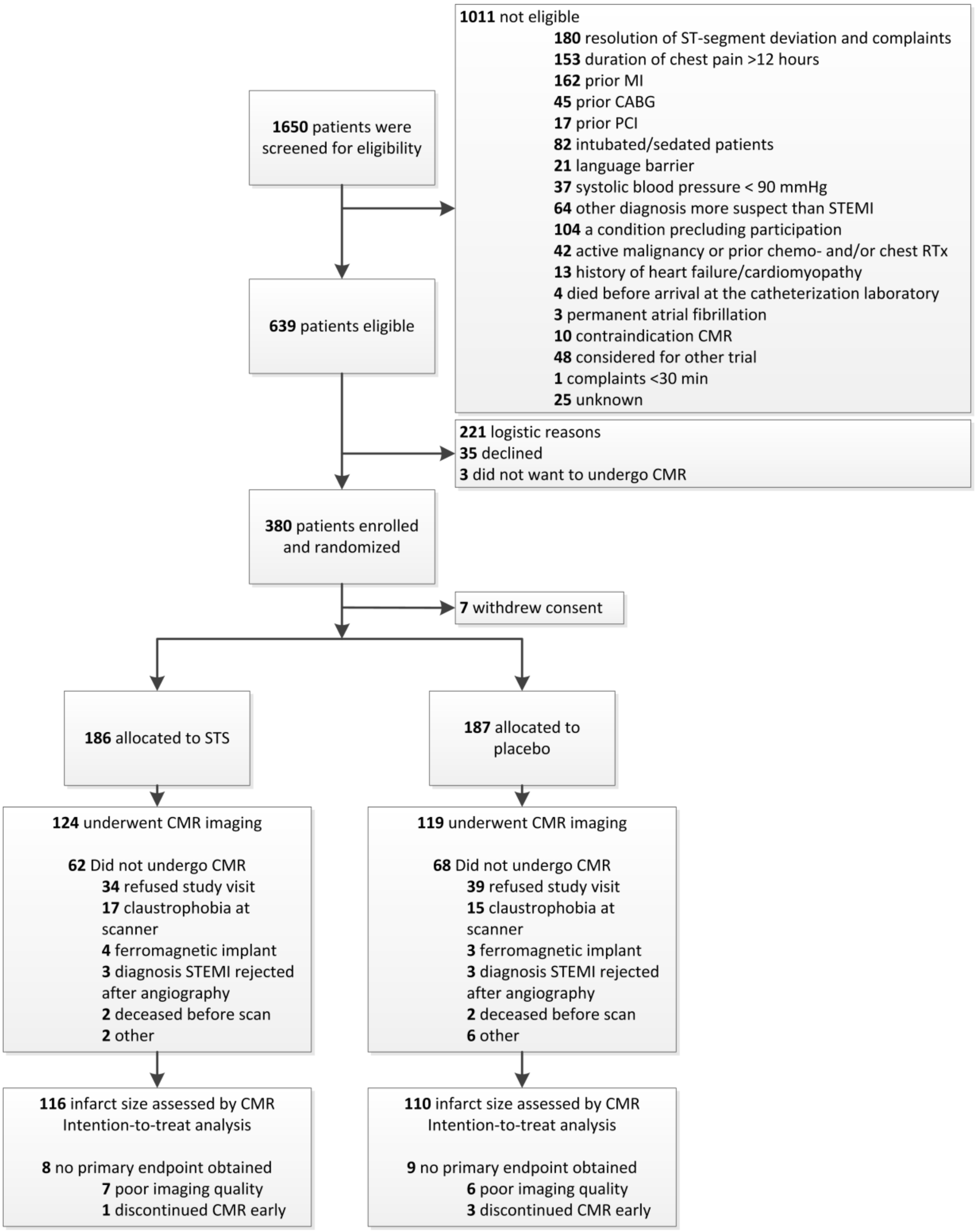


Abbreviations: CABG, coronary artery bypass graft; CMR, cardiac magnetic resonance imaging; MI, myocardial infarction; PCI percutaneous coronary intervention; STEMI, ST-elevation myocardial infarction; STS, sodium thiosulfate; RTx, radiotherapy.

**Supplemental Table 1 - Medication at discharge**

| **Medication at discharge** | **STS**<br>n=185* | **Placebo**<br>n=186* |
|---|---|---|
| Aspirin | 180 (97.3%) | 178 (95.7%) |
| Ticagrelor | 151 (81.6%) | 152 (81.7%) |
| Clopidogrel | 29 (15.7%) | 25 (13.4%) |
| Prasugrel | 1 (0.5%) | 0 |
| Beta-blocker | 168 (90.8%) | 168 (90.3%) |
| ACE-inhibitor or angiotensin receptor blocker | 153 (82.7%) | 158 (84.9%) |
| Statin | 177 (95.7%) | 179 (96.2%) |
| Calcium antagonist | 16 (8.6%) | 24 (12.9%) |
| Diuretics | 25 (13.5%) | 21 (11.3%) |
| Oral anticoagulants | | |
| Direct oral anticoagulants | 8 (4.3%) | 6 (3.2%) |
| Vitamin K antagonist | 4 (2.2%) | 5 (2.7%) |

*Two patients died during hospitalization (1 in STS group, 1 in placebo group)
Abbreviations: ACE, angiotensin converting enzyme; STS, sodium thiosulfate.

**Supplemental Table 2 - Baseline characteristics of the CMR population**

| Characteristic | STS<br>n =116 | Placebo<br>n =110 |
|---|---|---|
| **Demography** | | |
| Age at randomization (years), mean (SD) | 61.9 (10.7) | 59.1 (11.3) |
| Male sex, n (%) | 91 (78.4%) | 94 (85.5%) |
| Body Mass Index (kg/m$^2$), mean (SD) | 27.1 (3.9) | 27.2 (4.2) |
| Caucasian ethnicity, n (%) | 111 (95.7%) | 107 (97.3%) |
| **Prior conditions, n (%)** | | |
| Hypertension | 53 (45.7%) | 43 (39.1%) |
| Dyslipidemia | 35 (30.2%) | 37 (33.6%) |
| Current smokers | 38 (32.8%) | 39 (35.5%) |
| Positive family history | 48 (41.7%) | 51 (46.4%) |
| Diabetes Mellitus | 14 (12.1%) | 10 (9.1%) |
| Previous MI | 1 (0.9%) | 0 |
| Previous PCI | 2 (1.7%) | 1 (0.9%) |
| **Clinical characteristics** | | |
| Systolic blood pressure, mmHg, mean (SD) | 139 (26) | 143 (23) |
| Diastolic blood pressure, mmHg, mean (SD) | 83 (17) | 87 (15) |
| Heart rate, bpm, mean (SD) | 73 (17) | 75 (16) |
| Killip class I, n (%) | 106 (97.2%) | 107 (97.3%) |
| **Laboratory parameters, median [Q1, Q3]** | | |
| Hemoglobin (mmol/L) | 8.7 (8.1, 9.2) | 8.9 (8.3, 9.3) |
| Creatinine (µmol/L) | 74 (65, 84) | 78 (64, 86) |
| CK (U/L) | 135 (91, 208) | 144 (90, 242) |
| CK-MB activity (U/L) | 16 (13, 20) | 16 (13, 23) |
| NT-proBNP (ng/L) | 78 (39, 187) | 72 (33, 192) |
| Glucose (mmol/L) | 5.7 (5.5, 6.0) | 5.5 (5.4, 5.9) |

Baseline characteristics stratified by treatment allocation.
Abbreviations: CK, creatine kinase; CK-MB, creatine kinase myocardial band; CMR, cardiac magnetic resonance imaging; MI, myocardial infarction; NT-proBNP, N-terminal pro brain natriuretic peptide; PCI, percutaneous coronary intervention; Q1, 25th percentile; Q3, 75th percentile.

**Supplemental Table 3 - Procedural characteristics of the CMR population**

| **Characteristic** | **STS**<br>n =116 | **Placebo**<br>n =110 |
|---|---|---|
| Time from symptom onset to start study medication, (min), median [Q1, Q3] | 118 (77, 175) | 126 (90, 220) |
| Time from symptom onset to wire passage (min), median [Q1, Q3] | 134 (97, 178) | 142 (104, 244) |
| Single vessel disease, n (%) | 66 (56.9%) | 60 (54.5%) |
| Culprit territory, n (%) | | |
| Left anterior descending | 48 (41.4%) | 47 (42.7%) |
| Circumflex or marginal | 23 (19.8%) | 18 (16.4%) |
| Right coronary artery | 44 (37.9%) | 43 (39.1%) |
| Left main | 0 | 1 (0.9%) |
| No clear culprit | 1 (0.9%) | 1 (0.9%) |
| Medication from first medical care to PCI, n (%) | | |
| Aspirin | 116 (100%) | 109 (99.1%) |
| Loading dose of P2Y12 | 116 (100%) | 110 (100%) |
| Heparin | 116 (100%) | 110 (100%) |
| Glycoprotein IIb/IIIa inhibitor | 20 (17.2%) | 17 (15.5%) |
| TIMI flow grade pre-PCI, n (%) | | |
| 0 | 69 (59.5%) | 73 (66.4%) |
| 1 | 7 (6.0%) | 6 (5.5%) |
| 2 | 19 (16.4%) | 10 (9.1%) |
| 3 | 21 (18.1%) | 21 (19.1%) |
| Proximal lesion, n (%) | 44 (37.9%) | 43 (39.1%) |
| Initial intervention of culprit lesion, n (%) | | |
| PCI | 114 (98.3%) | 108 (98.2%) |
| CABG | 1 (0.9%) | 0 (0.0%) |
| Conservative | 1 (0.9%) | 2 (1.8%) |
| No reflow observed on angiography, n (%) | 3 (2.6%) | 5 (4.6%) |
| Distal embolization after PCI, n (%) | 10 (8.8%) | 5 (4.6%) |
| TIMI flow grade post-PCI, n (%) | | |
| 0 | 4 (3.5%) | 3 (2.8%) |
| 1 | 3 (2.6%) | 0 (0.0%) |
| 2 | 2 (1.8%) | 6 (5.6%) |
| 3 | 105 (92.1%) | 99 (91.7%) |

Abbreviations: CABG, coronary artery bypass graft; CMR, cardiac magnetic resonance imaging; PCI, percutaneous coronary intervention; Q1, 25th percentile; Q3, 75th percentile; TIMI, Thrombolysis in Myocardial Infarction.

**Supplemental Table 4 - Medication at discharge in the CMR population**

| Medication at discharge | STS<br>n=116 | Placebo<br>n=110 |
|---|---|---|
| Aspirin | 116 (100%) | 109 (99.1%) |
| Ticagrelor | 96 (82.8%) | 100 (90.9%) |
| Clopidogrel | 18 (15.5%) | 9 (8.2%) |
| Beta-blocker | 107 (92.2%) | 99 (90.0%) |
| ACE-inhibitor or angiotensin receptor blocker | 98 (84.5%) | 95 (86.4%) |
| Statin | 114 (98.3%) | 108 (98.2%) |
| Calcium antagonist | 7 (6.0%) | 9 (8.2%) |
| Diuretics | 12 (10.3%) | 8 (7.3%) |
| Oral anticoagulants | | |
| Direct oral anticoagulants | 4 (3.4%) | 2 (1.8%) |
| Vitamin K antagonist | 1 (0.9%) | 2 (1.8%) |

Abbreviations: ACE, angiotensin converting enzyme; CMR, cardiac magnetic resonance imaging; STS, sodium thiosulfate.

**Supplemental Table 5 - Per-protocol analysis**

| **Outcome** | **STS** | **Placebo** | ***P*-value** |
|---|---|---|---|
| Infarct size, % of LV mass, mean (SD) | 7.5 (6.5) | 8.8 (7.5) | 0.22* |

*Analyzed with Beta regression adjusted for site, treatment, and anterior MI location.
The Marginal average difference in infarct size was -1.1% (95% confidence interval -3.1% to 0.7%) for the STS group, compared with the placebo group.
Abbreviations: LV, left ventricle; SD, standard deviation; STS, sodium thiosulfate.
